# Successful Recanalization Using the Direct Aspiration First Pass Technique for Acute Carotid Stent Thrombosis Following Carotid Artery Stenting

**DOI:** 10.7759/cureus.86838

**Published:** 2025-06-27

**Authors:** Kosuke Ando, Takanori Suzuki, Takuya Moriwaki

**Affiliations:** 1 Department of Neurological Surgery, Asahi General Hospital, Chiba, JPN

**Keywords:** acute carotid stent thrombosis, carotid artery stenting, cerebral infarction, internal carotid artery stenosis, thrombectomy

## Abstract

Acute carotid stent thrombosis is a known serious complication of carotid artery stenting (CAS) with no established standard treatment. Here, we present such a case wherein early recanalization with mechanical thrombectomy proved effective. An 80-year-old man underwent CAS for symptomatic left internal carotid artery stenosis. On postoperative day 4, he developed right hemiparesis and aphasia. Cranial imaging revealed a left cerebral infarction and left carotid stent thrombosis. Mechanical thrombectomy using the direct aspiration first pass technique was performed for the in-stent occlusion, resulting in successful recanalization. Due to a partial contrast filling defect within the stent after recanalization, 80 mg of ozagrel sodium was administered intravenously and prasugrel was delivered via a nasogastric tube. Subsequent angiography showed no re-occlusion or stenosis, and the procedure was completed. No recurrence of cerebral infarction was observed postoperatively, and the patient was transferred to a rehabilitation facility.

## Introduction

Carotid artery stenting (CAS) has become a standard treatment option for carotid artery stenosis, alongside carotid endarterectomy (CEA). One of the most serious complications of CAS is acute carotid stent thrombosis (ACST), a rare but potentially life-threatening event. The etiology of ACST includes systemic factors such as inadequate antiplatelet therapy and hypercoagulability, as well as local factors including plaque protrusion into the stent, insufficient post-dilation, and arterial dissection [1]. Although various treatment options have been reported, including antithrombotic therapy, thrombolysis, CEA, and endovascular interventions, a definitive management strategy has not yet been established [2]. In recent years, with the advancement of endovascular techniques and devices, mechanical thrombectomy has emerged as a promising option, with increasing reports of successful recanalization [1,3]. Here, we present a case of ACST occurring in the acute phase after CAS, which was successfully treated with mechanical thrombectomy, resulting in a favorable clinical outcome.

## Case presentation

**Figure 1:**
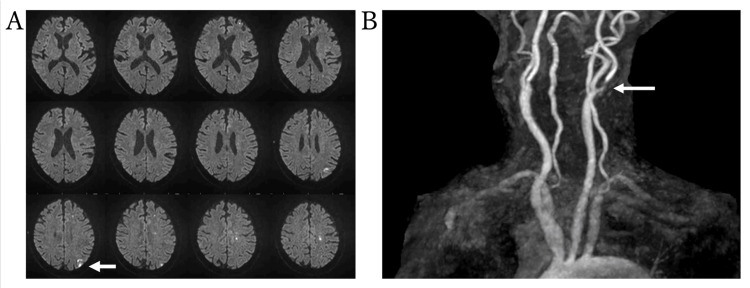
Acute infarction and carotid stenosis. Diffusion-weighted imaging shows a watershed infarction in the left hemisphere (arrow; A). MR angiography (MRA) reveals severe stenosis of the left internal carotid artery (arrow; B).

The patient was an 80-year-old man with a medical history of hypertension and dyslipidemia. He was transported to the emergency department of our hospital with gait disturbance and right hemiparesis. Cranial magnetic resonance imaging (MRI) revealed a left acute cerebral infarction (Figure 1A) and left internal carotid artery stenosis (Figure 1B). Acute cerebral infarction was managed according to the established treatment protocol. CAS for the symptomatic internal carotid artery stenosis was scheduled on day 28 of hospitalization.

Laboratory examination revealed no coagulation abnormalities, and antiplatelet medication efficacy was optimal (Table 1). Cervical MR angiography (MRA) showed severe stenoses of the left cervical internal carotid artery, and cranial MRI revealed a subacute cerebral infarction in the left watershed area. Cervical ultrasound examination revealed a peak systolic velocity of 700 cm/sec in the left internal carotid artery, indicating severe stenosis with heterogeneous echogenicity. Cervical plaque lesions demonstrated high signal intensity on T1-weighted imaging and 3D time-of-flight MRA, suggestive of vulnerable plaque.

**Table 1:** Results of coagulation and platelet function testing. All values were within normal limits. Platelet reactivity was assessed using the VerifyNow assay. PT-INR: prothrombin time-international normalized ratio, APTT: activated partial thromboplastin time, ARU: aspirin reaction units, PRU: P2Y12 reaction units.

Parameter	Actual Value	Reference Range	Unit
PT-INR	0.98	0.80-1.20	sec
APTT	26.0	25-35	sec
ARU	435	<550	ARU
PRU	187	160-200	PRU

**Figure 2:**
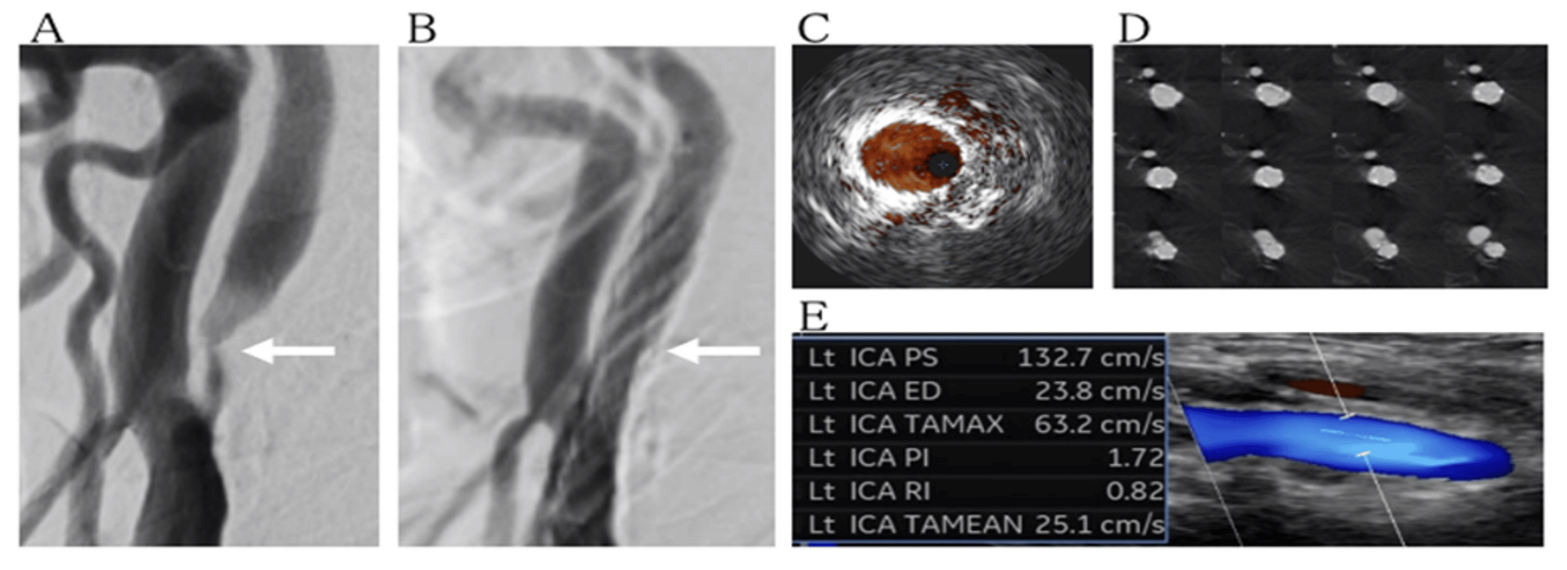
Pre- and post-stenting imaging of the left internal carotid artery. Lateral angiography reveals severe stenosis of the left internal carotid artery (arrow; A). Post-stenting angiography confirms good coverage and no residual stenosis (arrow; B). Intravascular ultrasound reveals a minimum lumen diameter >3 mm (C). 3D angiography shows no plaque protrusion (D). Postoperative carotid ultrasound shows no intrastent plaque and improved flow velocity (E). Lt: left, ICA: internal carotid artery, PS: peak systole, ED: end diastole, TAMAX: time-averaged maximum velocity, PI: pulsatility index, RI: resistance index, TAMEAN: time-averaged mean velocity.

Dual antiplatelet therapy with biaspirin (100 mg) and clopidogrel (75 mg) was continued, and optimal drug efficacy was confirmed by VerifyNow (IL Japan Co., Ltd., Tokyo, Japan) testing on the day before the procedure. CAS was performed under local anesthesia. After intravenous administration of 6,000 units of heparin, an 8Fr Optimo guiding catheter (Tokai, Aichi, Japan) was guided to the left common carotid artery via the femoral artery. Under distal protection with Spider 5.0 mm (Medtronic, MI, USA), pre-dilation was performed with a Shiden 3.0 mm × 20 mm balloon (Kaneka Medics, Osaka, Japan) at nominal pressure. A Casper Rx stent (8.0 mm × 20 mm; Terumo Corp., Tokyo, Japan) was placed from the internal carotid artery to the common carotid artery to sufficiently cover the stenotic area. Post-dilation was performed with Rx Genity 4.5 mm × 30 mm at nominal pressure (Figures 2A, 2B).

Intravascular ultrasound confirmed that the minimum lumen diameter at the site of greatest stenosis was more than 3 mm (Figure 2C) and that there was no plaque protrusion (Figure 2D). The procedure was completed after confirming satisfactory dilation of the lesion. Postoperatively, there were no new neurological symptoms apart from the sequelae of the previous cerebral infarction, and imaging revealed no intracranial lesions associated with the procedure. Postoperative carotid ultrasonography revealed no intrastent plaque, and blood velocity had improved compared to preoperative measurements (Figure 2E).

**Figure 3:**
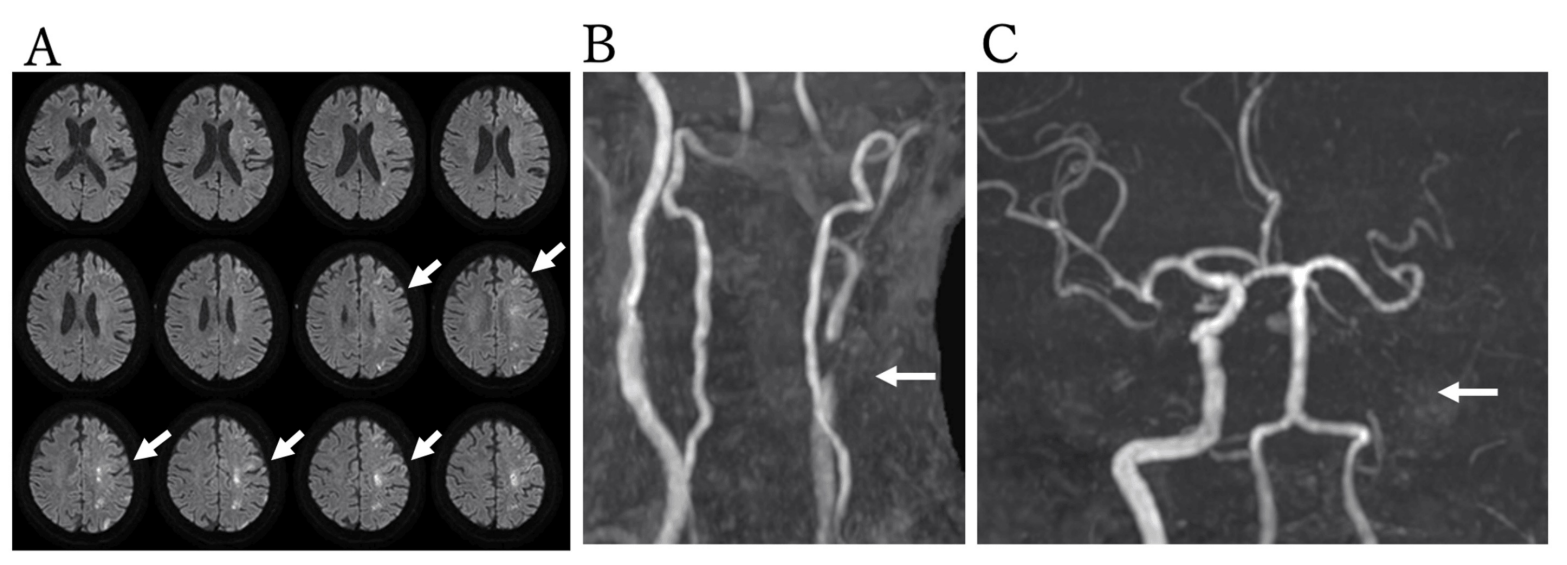
Ischemic lesions and carotid occlusion at stroke onset. MRI shows scattered infarctions in the left cerebral cortex (arrows; A). MR angiography (MRA) reveals occlusion of the left internal carotid artery (arrow; B, C).

On day 4 after CAS, the patient developed right hemiparesis and aphasia. Cranial MRI revealed scattered ischemic lesions in the left cerebral cortex (Figure 3A), while MRA demonstrated occlusion of the left internal carotid artery (Figures 3B, 3C). Mechanical thrombectomy was performed to prevent progression of cerebral ischemia.

**Figure 4:**
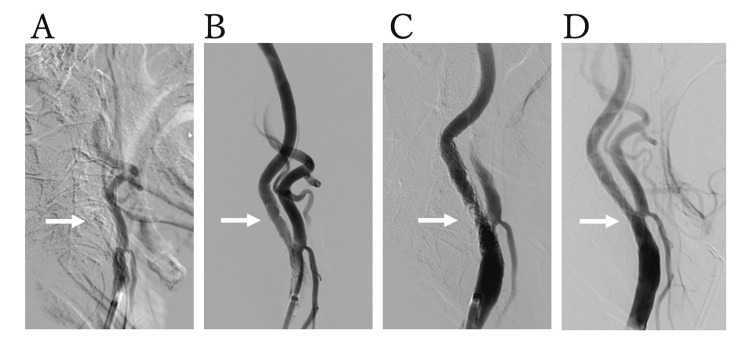
Endovascular treatment for in-stent occlusion of the left internal carotid artery. Angiography shows in-stent occlusion of the left internal carotid artery (arrow; A). Mechanical thrombectomy with a React 71 catheter achieves recanalization (arrow; B). Follow-up angiography reveals a contrast filling defect at the stent’s proximal end (arrow; C). Final angiogram confirms no residual stenosis or occlusion (arrow; D).

Endovascular treatment was performed with an 8Fr Optimo balloon-guiding catheter placed in the left common carotid artery via the femoral artery. Cerebral angiography showed an in-stent occlusion (Figure 4A). The Optimo catheter was advanced to the origin of the lesion, and direct manual aspiration was performed from the guiding catheter. The red thrombus was retrieved, but complete recanalization was not achieved. We decided to aspirate the in-stent thrombus using a React 71 (Medtronic, MI, US) catheter. The React 71 catheter was slowly advanced from the proximal to the distal end of the stent, and while applying manual aspiration, it was retrieved into the guiding catheter, successfully removing a large amount of thrombus. Angiography of the lesion site confirmed antegrade flow (Figure 4B). After a brief waiting period, cervical angiography was performed to assess the risk of re-occlusion, which revealed a contrast filling defect at the proximal end of the carotid stent (Figure 4C). To enhance antiplatelet therapy, ozagrel sodium (80 mg) was administered intravenously, and prasugrel (20 mg) was delivered via a nasogastric tube. The aspiration catheter was again advanced to the proximal end of the stent, and manual aspiration thrombectomy was performed. Repeat angiography showed resolution of the filling defect with no occlusion, and the procedure was completed (Figure 4D).

Postoperatively, dual antiplatelet therapy was changed to biaspirin and prasugrel (3.75 mg), with the addition of heparin at a dose of 20,000 units/day. On day 5 after CAS, digital subtraction angiography was performed, which showed no findings suggestive of in-stent plaque. Heparin was gradually tapered and discontinued on day 7. Follow-up cranial imaging showed no new cerebral infarction, and carotid evaluation showed no evidence of restenosis or occlusion. The patient was transferred to a rehabilitation facility on day 81 of hospitalization with residual aphasia and right hemiparesis.

## Discussion

ACST is a rare but serious complication of carotid artery stenting (CAS), occurring in 0.2%-0.8% of cases, most commonly within the first week post-procedure [1]. Early recanalization is essential to prevent irreversible cerebral ischemia, yet no standardized treatment strategy has been established [2]. Commonly reported etiologies include inadequate antiplatelet therapy, hypercoagulability, in-stent plaque protrusion, vasospasm, insufficient post-dilation, and vessel dissection [1,4-6]. Among these, suboptimal antiplatelet therapy is the most frequent. Clopidogrel resistance, often due to CYP2C19 loss-of-function polymorphisms, especially prevalent in East Asian populations, has been associated with periprocedural ischemic events [7]. Hypercoagulability due to malignancy has also been implicated in rare cases [8].

In this case, preprocedural platelet function testing using the VerifyNow-P2Y12 assay indicated an adequate antiplatelet response. The patient had no significant comorbidities aside from diabetes mellitus and no abnormalities in coagulation tests or evidence of systemic thrombophilia. Intraoperative angiography showed no local procedural issues, such as dissection, insufficient dilation, or plaque protrusion. Although acute in-stent thrombosis has been reported in cases involving dual-layer stents such as the CASPER Rx, most occurred during the acute phase of cerebral infarction, when the antiplatelet effect may be insufficient and the stent’s structural properties may increase thrombogenicity [9].

To our knowledge, this is a rare instance of ACST in a patient without identifiable systemic or procedural risk factors. One possible contributing factor is the limited reproducibility of platelet function testing. The VerifyNow assay has shown intra-individual variability, and repeated testing may improve accuracy [10]. In this case, additional testing in the periprocedural period may have yielded different results. Furthermore, prasugrel, unlike clopidogrel, is not affected by CYP2C19 polymorphisms and provides more potent and consistent platelet inhibition [11]. No recurrent thrombotic events occurred after switching to prasugrel in this case, suggesting its superiority in this patient and supporting its use in similar clinical scenarios.

No definitive prophylactic strategies for ACST currently exist. However, it has been recommended that at least two carotid duplex ultrasonography exams be performed within the first week after CAS [1,12]. This facilitates early detection of in-stent thrombosis and timely initiation of anticoagulation, potentially halting thrombus progression. In our case, ACST occurred on postoperative day 4, but only one ultrasound examination had been performed. Additional imaging might have enabled earlier intervention.

Treatment options for ACST include intensified anticoagulation, thrombolytic therapy (recombinant tissue plasminogen activator (rtPA) or urokinase), mechanical thrombectomy, angioplasty or repeat stenting, and surgical procedures such as stent removal or carotid endarterectomy [6,12-15]. In this case, mechanical thrombectomy was selected to achieve early recanalization.

Mechanical thrombectomy techniques include A Direct Aspiration First Pass Technique (ADAPT), which uses an aspiration catheter alone, and the combined technique, which also uses a stent retriever. Both are effective for treating acute in-stent thrombosis [3]. However, stent retriever treatment carries risks of entanglement with the carotid stent, endothelial damage, and distal embolism caused by passing through the distal lesion. Therefore, when feasible, treatment with an aspiration catheter alone, as in our case, is theoretically preferable.

## Conclusions

We reported a case of cerebral infarction due to ACST after CAS. ACST often follows a serious clinical course, and prompt treatment is necessary to prevent progression of cerebral infarction. Mechanical thrombectomy, which allows for rapid intervention, can be considered an appropriate option for reperfusion therapy. Among the available techniques, ADAPT is preferable due to its lower risk of endothelial damage and distal embolism.
